# 4-Methyl­anilinium 4-hy­droxy­benzene­sulfonate

**DOI:** 10.1107/S1600536813009410

**Published:** 2013-04-13

**Authors:** J. V. Jovita, S. Sathya, G. Usha, R. Vasanthi, P. Sagayaraj

**Affiliations:** a PG and Research Department of Physics, Queen Mary’s College, Chennai-4, Tamilnadu, India; b Department of Physics, Loyola college, Chennai-34, Tamilnadu, India

## Abstract

In the crystal of the title molecular salt, C_7_H_10_N^+^·C_6_H_5_O_4_S^−^, the benzene­sulfonate units are linked through phenol–sulfonate O—H⋯O hydrogen bonds, forming chains along the *c*-axis direction. These chains are linked *via* N—H⋯O hydrogen bonds involving two of the three H atoms of the ammonium group of the 4-methyl­anilium cation, giving rise to two-dimensional networks parallel to the *bc* plane which are further connected through an additional N—H⋯O inter­action in which the third ammonium H atom is involved, generating a three-dimensional network.

## Related literature

For the biological activity of related compounds, see: Fukami *et al.* (2000 ▶). For standard bond lengths, see: Allen *et al.* (1987 ▶). 
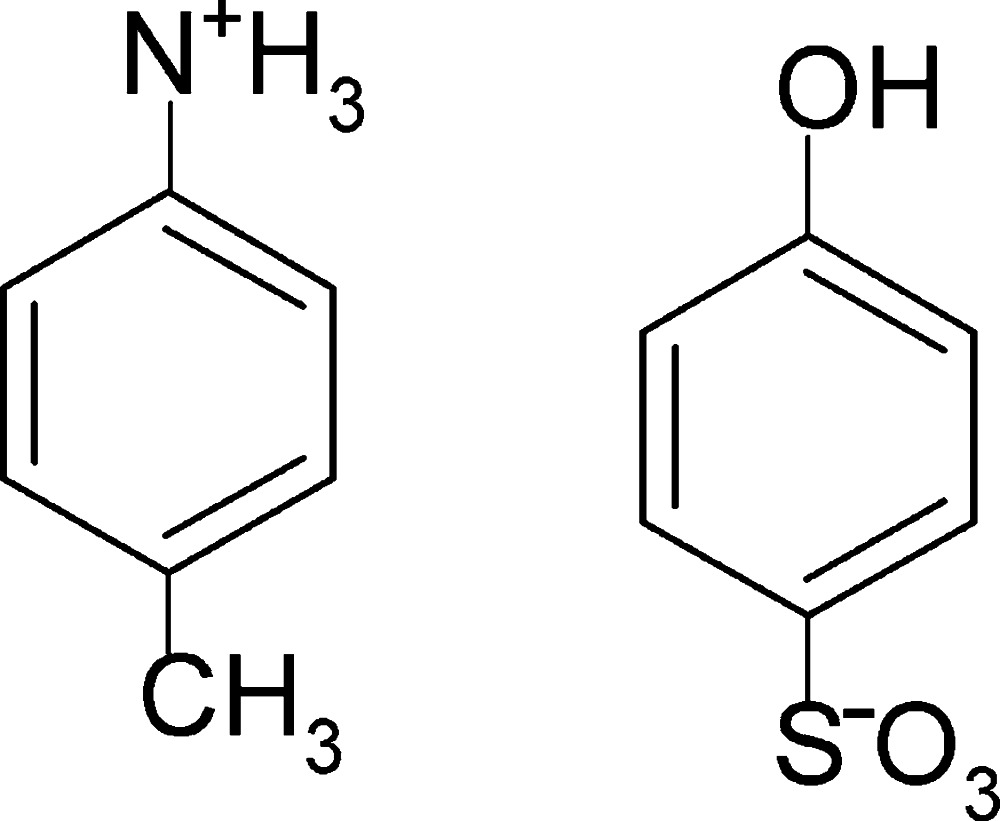


## Experimental

### 

#### Crystal data

C_7_H_10_N^+^·C_6_H_5_O_4_S^−^
*M* *_r_* = 281.32
Monoclinic, 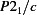

*a* = 11.6450 (2) Å
*b* = 7.1670 (1) Å
*c* = 16.3080 (3) Å
β = 107.654 (1)°
*V* = 1296.96 (4) Å^3^
*Z* = 4
Mo *K*α radiation
μ = 0.26 mm^−1^
*T* = 293 K
0.30 × 0.30 × 0.20 mm

#### Data collection

Bruker Kappa APEXII CCD diffractometer
Absorption correction: multi-scan (*SADABS*; Bruker, 2004 ▶) *T*_min_ = 0.926, *T*_max_ = 0.950
11047 measured reflections
2285 independent reflections
2045 reflections with *I* > 2σ(*I*)
*R*_int_ = 0.029

#### Refinement

*R*[*F* ^2^ > 2σ(*F* ^2^)] = 0.030
*wR*(*F* ^2^) = 0.091
*S* = 1.06
2285 reflections
175 parameters
H-atom parameters constrained
Δρ_max_ = 0.24 e Å^−3^
Δρ_min_ = −0.29 e Å^−3^

### 

Data collection: *APEX2* (Bruker, 2004 ▶); cell refinement: *APEX2* and *SAINT* (Bruker, 2004 ▶); data reduction: *SAINT* and *XPREP* (Bruker, 2004 ▶); program(s) used to solve structure: *SIR92* (Altomare *et al.*, 1993 ▶); program(s) used to refine structure: *SHELXL97* (Sheldrick, 2008 ▶); molecular graphics: *ORTEP-3 for Windows* (Farrugia, 2012 ▶); software used to prepare material for publication: *PLATON* (Spek, 2009 ▶).

## Supplementary Material

Crystal structure: contains datablock(s) I, global. DOI: 10.1107/S1600536813009410/lr2102sup1.cif

Structure factors: contains datablock(s) I. DOI: 10.1107/S1600536813009410/lr2102Isup2.hkl

Supplementary material file. DOI: 10.1107/S1600536813009410/lr2102Isup3.cml

Additional supplementary materials:  crystallographic information; 3D view; checkCIF report

## Figures and Tables

**Table 1:** Hydrogen-bond geometry (Å, °)

*D*—H⋯*A*	*D*—H	H⋯*A*	*D*⋯*A*	*D*—H⋯*A*
N1—H1*C*⋯O1^i^	0.89	1.96	2.8367 (19)	169
N1—H1*B*⋯O4^ii^	0.89	1.96	2.839 (2)	170
N1—H1*A*⋯O2^iii^	0.89	1.94	2.8091 (19)	166
O4—H4⋯O3^iii^	0.82	1.82	2.6343 (17)	173

Symmetry codes: (i) 
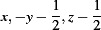
; (ii) 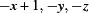; (iii) 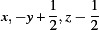.

**Fig. 1.**
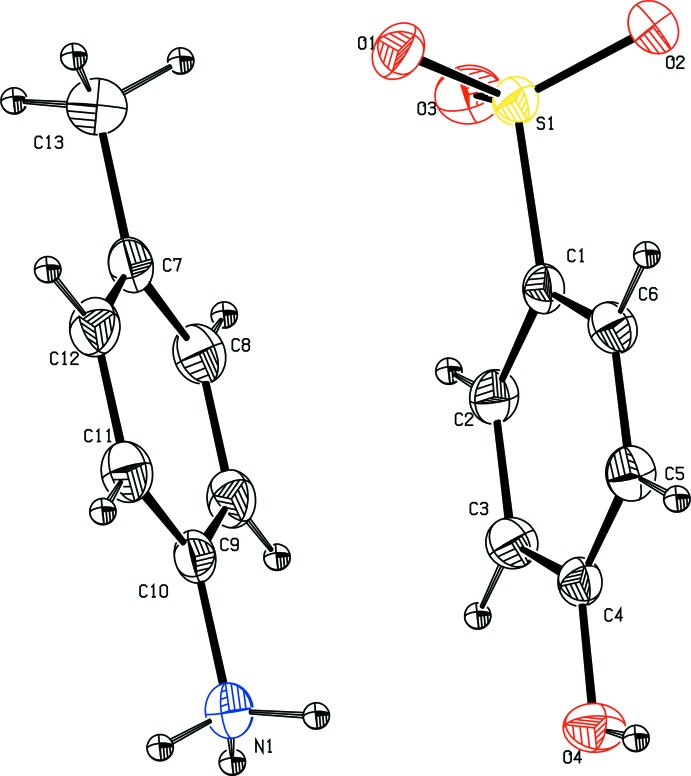
The molecular structure of the title compound, with displacement ellipsoids drawn at the 30% probability level.

**Fig. 2.**
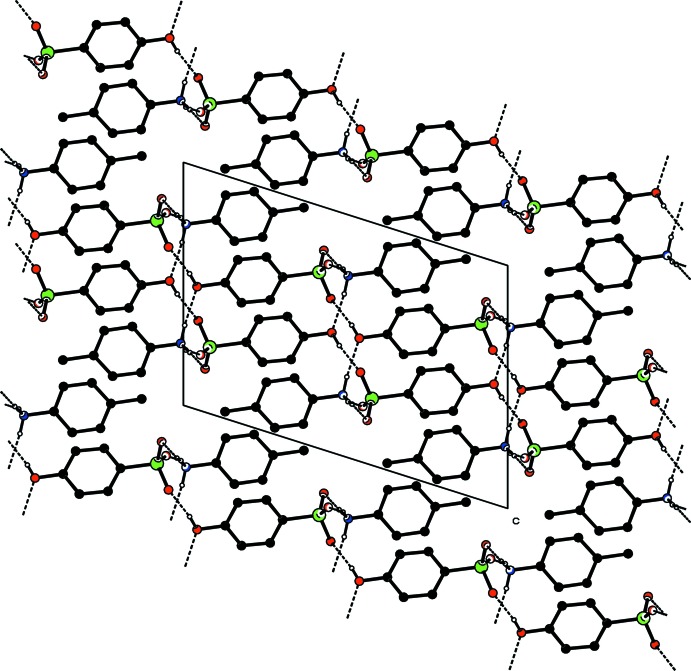
A view of the the packing of the the title compound. Dashed lines indicate hydrogen bonds.

## supplementary crystallographic information

### Comment

The stucture of the title compound, (I), is shown in Figure 1. Bond lengh and angles are within the standard values (Allen *et al.*, 1987). In the crystal the phenolsulfonate units are joined together through O4-H4···O3 hydrogen bonds, forming chains along the *c* crystallographic axis. These chains are subsequently linked together through N-H···O interactions involving the 4-methylanilium units. First, giving rise to a two-dimensional network parallel to *bc* plane, through interactions involving two of the three hydrogens of the ammonium moiety: N1-H1A···O2 and N1-H1C···O1 and finally generating a three-dimensional network through the use of the third hydrogen atom bonded to the nitrogen: N1-H1B···O4 (Table 1 and Figure 2).

### Experimental

The 4-MAPS compound was synthesized by the reaction of equimolar mixture of 4-methyl aniline and phenolsulfonic acid. To a saturated solution of 4-methyl aniline in acetone, phenolsulfonic acid was slowly added at room temperature. The solution was stirred for six hours to get an homogeneous solution, filtered and kept for slow evaporation at room temperature. After that a saturated solution of 4-MAPS was prepared by using methanol at room temperature. The prepared solution was kept to constant temperature in water bath at 30° C to avoid the effect of fluctuation in room temperature. An slow evaporation process was allowed for a period of 15 days. The grown crystals of an approximate size of 12 *x* 9 *x* 2 mm3 were harvested and re-crystallized to grow pure crystals for further studies.

### Refinement

H atoms were positioned geometrically and treated as riding on their parent atoms, with C—H distance of 0.93 - 0.96 Å, N—H distance of 0.89 Å, O—H distance of 0.82 Å and *U*_iso_(H) = 1.2Ueq(N and C_aromatic_) and *U*_iso_(H) = 1.5Ueq( C_methyl_)

### Crystal data

**Table:**

C_7_H_10_N^+^·C_6_H_5_O_4_S^−^	*F*(000) = 592
*M**_r_* = 281.32	*D*_x_ = 1.441 Mg m^−^^3^
Monoclinic, *P*2_1_/*c*	Mo *K*α radiation, λ = 0.71073 Å
Hall symbol: -P 2ybc	Cell parameters from 5614 reflections
*a* = 11.6450 (2) Å	θ = 2.8–29.4°
*b* = 7.1670 (1) Å	µ = 0.26 mm^−^^1^
*c* = 16.3080 (3) Å	*T* = 293 K
β = 107.654 (1)°	Block, colourless
*V* = 1296.96 (4) Å^3^	0.30 × 0.30 × 0.20 mm
*Z* = 4	

### Data collection

**Table:**

Bruker Kappa APEXII CCD diffractometer	2285 independent reflections
Radiation source: fine-focus sealed tube	2045 reflections with *I* > 2σ(*I*)
Graphite monochromator	*R*_int_ = 0.029
ω and φ scan	θ_max_ = 25.0°, θ_min_ = 2.6°
Absorption correction: multi-scan (*SADABS*; Bruker, 2004)	*h* = −13→13
*T*_min_ = 0.926, *T*_max_ = 0.950	*k* = −8→8
11047 measured reflections	*l* = −19→19

### Refinement

**Table:**

Refinement on *F*^2^	Secondary atom site location: difference Fourier map
Least-squares matrix: full	Hydrogen site location: inferred from neighbouring sites
*R*[*F*^2^ > 2σ(*F*^2^)] = 0.030	H-atom parameters constrained
*wR*(*F*^2^) = 0.091	*w* = 1/[σ^2^(*F*_o_^2^) + (0.0467*P*)^2^ + 0.4532*P*] where *P* = (*F*_o_^2^ + 2*F*_c_^2^)/3
*S* = 1.06	(Δ/σ)_max_ < 0.001
2285 reflections	Δρ_max_ = 0.24 e Å^−^^3^
175 parameters	Δρ_min_ = −0.29 e Å^−^^3^
0 restraints	Extinction correction: *SHELXL97* (Sheldrick, 2008), Fc^*^=kFc[1+0.001xFc^2^λ^3^/sin(2θ)]^-1/4^
Primary atom site location: structure-invariant direct methods	Extinction coefficient: 0.0070 (12)

### Special details

**Table:**

Geometry. All e.s.d.'s (except the e.s.d. in the dihedral angle between two l.s. planes) are estimated using the full covariance matrix. The cell e.s.d.'s are taken into account individually in the estimation of e.s.d.'s in distances, angles and torsion angles; correlations between e.s.d.'s in cell parameters are only used when they are defined by crystal symmetry. An approximate (isotropic) treatment of cell e.s.d.'s is used for estimating e.s.d.'s involving l.s. planes.
Refinement. Refinement of *F*^2^ against ALL reflections. The weighted *R*-factor *wR* and goodness of fit *S* are based on *F*^2^, conventional *R*-factors *R* are based on *F*, with *F* set to zero for negative *F*^2^. The threshold expression of *F*^2^ > σ(*F*^2^) is used only for calculating *R*-factors(gt) *etc*. and is not relevant to the choice of reflections for refinement. *R*-factors based on *F*^2^ are statistically about twice as large as those based on *F*, and *R*- factors based on ALL data will be even larger.

### Fractional atomic coordinates and isotropic or equivalent isotropic displacement parameters (Å^2^)

**Table:**

	*x*	*y*	*z*	*U*_iso_*/*U*_eq_	
C1	0.65813 (14)	0.2000 (2)	0.30612 (10)	0.0311 (4)	
C2	0.54731 (14)	0.1192 (2)	0.26762 (10)	0.0375 (4)	
H2	0.5057	0.0616	0.3011	0.045*	
C3	0.49842 (14)	0.1242 (2)	0.17924 (10)	0.0391 (4)	
H3	0.4240	0.0688	0.1532	0.047*	
C4	0.55951 (14)	0.2109 (2)	0.12920 (10)	0.0328 (4)	
C5	0.67033 (15)	0.2933 (2)	0.16791 (11)	0.0365 (4)	
H5	0.7116	0.3521	0.1345	0.044*	
C6	0.71913 (14)	0.2878 (2)	0.25594 (10)	0.0355 (4)	
H6	0.7935	0.3432	0.2820	0.043*	
C7	0.91246 (16)	−0.3025 (2)	0.27651 (11)	0.0420 (4)	
C8	0.79646 (16)	−0.2324 (3)	0.24420 (12)	0.0455 (4)	
H8	0.7542	−0.2001	0.2822	0.055*	
C9	0.74260 (15)	−0.2096 (2)	0.15731 (12)	0.0423 (4)	
H9	0.6646	−0.1628	0.1367	0.051*	
C10	0.80527 (14)	−0.2568 (2)	0.10108 (11)	0.0353 (4)	
C11	0.92034 (15)	−0.3268 (2)	0.13049 (12)	0.0432 (4)	
H11	0.9623	−0.3581	0.0922	0.052*	
C12	0.97244 (15)	−0.3499 (3)	0.21799 (12)	0.0450 (4)	
H12	1.0500	−0.3986	0.2382	0.054*	
C13	0.9715 (2)	−0.3262 (3)	0.37143 (13)	0.0614 (6)	
H13A	0.9581	−0.2166	0.4011	0.092*	
H13B	1.0565	−0.3446	0.3825	0.092*	
H13C	0.9377	−0.4327	0.3914	0.092*	
N1	0.74899 (13)	−0.2321 (2)	0.00829 (9)	0.0424 (4)	
H1A	0.7692	−0.1211	−0.0076	0.064*	
H1B	0.6692	−0.2387	−0.0039	0.064*	
H1C	0.7743	−0.3214	−0.0200	0.064*	
O1	0.82148 (11)	0.05029 (17)	0.43307 (7)	0.0458 (3)	
O2	0.77002 (13)	0.37156 (18)	0.44678 (8)	0.0559 (4)	
O3	0.63246 (11)	0.1221 (2)	0.45415 (8)	0.0526 (4)	
O4	0.50641 (11)	0.21139 (18)	0.04252 (7)	0.0450 (3)	
H4	0.5499	0.2661	0.0192	0.068*	
S1	0.72587 (4)	0.18720 (6)	0.41863 (2)	0.03519 (16)	

### Atomic displacement parameters (Å^2^)

**Table:**

	*U*^11^	*U*^22^	*U*^33^	*U*^12^	*U*^13^	*U*^23^
C1	0.0357 (8)	0.0251 (8)	0.0341 (8)	0.0030 (6)	0.0128 (7)	−0.0003 (6)
C2	0.0401 (9)	0.0344 (9)	0.0402 (9)	−0.0053 (7)	0.0156 (7)	0.0051 (7)
C3	0.0354 (8)	0.0381 (9)	0.0416 (9)	−0.0091 (7)	0.0084 (7)	0.0018 (7)
C4	0.0377 (8)	0.0283 (8)	0.0332 (8)	0.0027 (6)	0.0119 (7)	−0.0009 (6)
C5	0.0384 (9)	0.0356 (9)	0.0404 (9)	−0.0032 (7)	0.0193 (7)	0.0013 (7)
C6	0.0329 (8)	0.0336 (9)	0.0405 (9)	−0.0048 (7)	0.0119 (7)	−0.0026 (7)
C7	0.0459 (9)	0.0283 (9)	0.0484 (10)	−0.0016 (7)	0.0093 (8)	−0.0028 (7)
C8	0.0492 (10)	0.0398 (10)	0.0502 (10)	0.0040 (8)	0.0192 (8)	−0.0065 (8)
C9	0.0358 (9)	0.0345 (9)	0.0548 (11)	0.0065 (7)	0.0108 (8)	−0.0039 (8)
C10	0.0376 (8)	0.0243 (8)	0.0429 (9)	−0.0023 (7)	0.0105 (7)	−0.0014 (7)
C11	0.0404 (9)	0.0381 (9)	0.0542 (11)	0.0022 (7)	0.0191 (8)	−0.0033 (8)
C12	0.0349 (9)	0.0372 (9)	0.0589 (11)	0.0045 (7)	0.0079 (8)	0.0003 (8)
C13	0.0728 (14)	0.0525 (12)	0.0495 (12)	0.0006 (10)	0.0045 (10)	−0.0017 (9)
N1	0.0475 (8)	0.0328 (8)	0.0455 (8)	−0.0036 (6)	0.0119 (7)	0.0014 (6)
O1	0.0507 (7)	0.0420 (7)	0.0437 (7)	0.0128 (6)	0.0126 (5)	0.0063 (5)
O2	0.0760 (9)	0.0356 (7)	0.0452 (7)	−0.0002 (7)	0.0020 (6)	−0.0077 (6)
O3	0.0587 (8)	0.0666 (9)	0.0394 (7)	0.0015 (7)	0.0252 (6)	0.0000 (6)
O4	0.0464 (7)	0.0555 (8)	0.0325 (6)	−0.0069 (6)	0.0110 (5)	−0.0001 (5)
S1	0.0436 (3)	0.0304 (2)	0.0315 (2)	0.00412 (16)	0.01122 (17)	−0.00084 (15)

### Geometric parameters (Å, º)

**Table:**

C1—C2	1.380 (2)	C9—C10	1.376 (2)
C1—C6	1.386 (2)	C9—H9	0.9300
C1—S1	1.7662 (16)	C10—C11	1.374 (2)
C2—C3	1.380 (2)	C10—N1	1.466 (2)
C2—H2	0.9300	C11—C12	1.381 (3)
C3—C4	1.382 (2)	C11—H11	0.9300
C3—H3	0.9300	C12—H12	0.9300
C4—O4	1.3603 (19)	C13—H13A	0.9600
C4—C5	1.385 (2)	C13—H13B	0.9600
C5—C6	1.375 (2)	C13—H13C	0.9600
C5—H5	0.9300	N1—H1A	0.8900
C6—H6	0.9300	N1—H1B	0.8900
C7—C12	1.385 (3)	N1—H1C	0.8900
C7—C8	1.387 (2)	O1—S1	1.4487 (12)
C7—C13	1.501 (3)	O2—S1	1.4414 (13)
C8—C9	1.374 (3)	O3—S1	1.4556 (13)
C8—H8	0.9300	O4—H4	0.8200
			
C2—C1—C6	119.85 (15)	C11—C10—N1	119.19 (15)
C2—C1—S1	120.82 (12)	C9—C10—N1	119.79 (15)
C6—C1—S1	119.30 (12)	C10—C11—C12	118.74 (16)
C1—C2—C3	119.81 (15)	C10—C11—H11	120.6
C1—C2—H2	120.1	C12—C11—H11	120.6
C3—C2—H2	120.1	C11—C12—C7	121.83 (16)
C2—C3—C4	120.32 (15)	C11—C12—H12	119.1
C2—C3—H3	119.8	C7—C12—H12	119.1
C4—C3—H3	119.8	C7—C13—H13A	109.5
O4—C4—C3	117.48 (14)	C7—C13—H13B	109.5
O4—C4—C5	122.66 (14)	H13A—C13—H13B	109.5
C3—C4—C5	119.85 (15)	C7—C13—H13C	109.5
C6—C5—C4	119.78 (15)	H13A—C13—H13C	109.5
C6—C5—H5	120.1	H13B—C13—H13C	109.5
C4—C5—H5	120.1	C10—N1—H1A	109.5
C5—C6—C1	120.38 (14)	C10—N1—H1B	109.5
C5—C6—H6	119.8	H1A—N1—H1B	109.5
C1—C6—H6	119.8	C10—N1—H1C	109.5
C12—C7—C8	117.63 (17)	H1A—N1—H1C	109.5
C12—C7—C13	120.91 (17)	H1B—N1—H1C	109.5
C8—C7—C13	121.46 (17)	C4—O4—H4	109.5
C9—C8—C7	121.47 (17)	O2—S1—O1	112.76 (8)
C9—C8—H8	119.3	O2—S1—O3	113.86 (8)
C7—C8—H8	119.3	O1—S1—O3	110.38 (8)
C8—C9—C10	119.31 (16)	O2—S1—C1	106.67 (7)
C8—C9—H9	120.3	O1—S1—C1	106.46 (7)
C10—C9—H9	120.3	O3—S1—C1	106.13 (7)
C11—C10—C9	121.02 (16)		
			
C6—C1—C2—C3	0.8 (2)	C8—C9—C10—C11	0.3 (3)
S1—C1—C2—C3	−177.26 (13)	C8—C9—C10—N1	−179.65 (15)
C1—C2—C3—C4	−0.5 (2)	C9—C10—C11—C12	0.2 (3)
C2—C3—C4—O4	−179.85 (15)	N1—C10—C11—C12	−179.88 (15)
C2—C3—C4—C5	0.0 (2)	C10—C11—C12—C7	−0.7 (3)
O4—C4—C5—C6	−179.95 (14)	C8—C7—C12—C11	0.8 (3)
C3—C4—C5—C6	0.2 (2)	C13—C7—C12—C11	−179.14 (17)
C4—C5—C6—C1	0.1 (2)	C2—C1—S1—O2	−134.51 (14)
C2—C1—C6—C5	−0.6 (2)	C6—C1—S1—O2	47.42 (14)
S1—C1—C6—C5	177.49 (12)	C2—C1—S1—O1	104.85 (14)
C12—C7—C8—C9	−0.3 (3)	C6—C1—S1—O1	−73.21 (14)
C13—C7—C8—C9	179.63 (17)	C2—C1—S1—O3	−12.76 (15)
C7—C8—C9—C10	−0.2 (3)	C6—C1—S1—O3	169.17 (13)

### Hydrogen-bond geometry (Å, º)

**Table:**

*D*—H···*A*	*D*—H	H···*A*	*D*···*A*	*D*—H···*A*
N1—H1*C*···O1^i^	0.89	1.96	2.8367 (19)	169
N1—H1*B*···O4^ii^	0.89	1.96	2.839 (2)	170
N1—H1*A*···O2^iii^	0.89	1.94	2.8091 (19)	166
O4—H4···O3^iii^	0.82	1.82	2.6343 (17)	173

Symmetry codes: (i) *x*, −*y*−1/2, *z*−1/2; (ii) −*x*+1, −*y*, −*z*; (iii) *x*, −*y*+1/2, *z*−1/2.
